# Vaping and socioeconomic inequalities in smoking cessation and relapse: a longitudinal analysis of the UK Household Longitudinal Study

**DOI:** 10.1136/tc-2022-057728

**Published:** 2023-04-11

**Authors:** Iain Hardie, Michael James Green

**Affiliations:** 1 MRC/CSO Social and Public Health Sciences Unit, School of Health and Wellbeing, University of Glasgow, Glasgow, UK; 2 Department of Psychology, School of Philosophy, Psychology and Language Sciences, The University of Edinburgh, Edinburgh, UK; 3 Department of Obstetrics and Gynecology, Duke University, Durham, North Carolina, USA

**Keywords:** Electronic nicotine delivery devices, Cessation, Socioeconomic status

## Abstract

**Background:**

Smoking is a key cause of socioeconomic health inequalities. Vaping is considered less harmful than smoking and has become a popular smoking cessation aid, and therefore has potential to reduce inequalities in smoking.

**Methods:**

We used longitudinal data from 25 102 participants in waves 8–10 (2016 to early 2020) of the UK Household Longitudinal Study to examine how vaping affects socioeconomic inequalities in smoking cessation and relapse. Marginal structural models were used to investigate whether vaping mediates or moderates associations between educational attainment and smoking cessation and relapse over time. Multiple imputation and weights were used to adjust for missing data.

**Results:**

Respondents without degrees were less likely to stop smoking than those with a degree (OR: 0.65; 95% CI 0.54–0.77), and more likely to relapse (OR: 1.74; 95% CI 1.37–2.22), but this inequality in smoking cessation was not present among regular vapers (OR: 0.99; 95% CI 0.54–1.82). Sensitivity analyses suggested that this finding did not hold when comparing those with or without any qualifications. Inequalities in smoking relapse did not clearly differ by vaping status.

**Conclusions:**

Vaping may be especially helpful as a cessation aid for smokers without degree level education and therefore may help reduce inequalities in smoking. Nevertheless, other supports or aids may be needed to reach the most disadvantaged (ie, those with no qualifications) and to help people avoid relapse after cessation, though we did not find clear evidence suggesting that vaping would increase inequalities in relapse.

**WHAT IS ALREADY KNOWN ON THIS TOPIC**

- Socioeconomic inequalities in smoking cessation have narrowed in recent years since e-cigarettes have become more widely available as a cessation aid.
- It is not clear whether this was as a result of increased vaping or other due to other confounding factors.
- Existing research on vaping and socioeconomic inequalities in smoking cessation has been limited to using cross-sectional data.

**WHAT THIS STUDY ADDS**

- Using longitudinal data, over 2 years of follow-up, our study suggests that vaping may reduce socioeconomic inequalities in smoking cessation, as smoking cessation is less strongly associated with having degree level education among regular vapers.
- However, sensitivity analyses suggested that this finding did not hold when comparing those with or without any qualifications.
- We did not find clear evidence to suggest that vaping would adversely affect inequalities in smoking relapse.

**HOW THIS STUDY MIGHT AFFECT RESEARCH, PRACTICE OR POLICY**

- Vaping regulations should consider that it may have a net positive impact on inequalities in smoking cessation, without adversely impacting on inequalities in smoking relapse.
- However, other aids may still be needed for the most disadvantaged and to help people avoid smoking relapse.

## Introduction

 Smoking is a leading cause of ill health and contributes substantially to socioeconomic health inequalities.^1^,^4^ E-cigarettes (ie, vaping products) offer an alternative nicotine delivery method to smoking. They are currently the most popular smoking cessation aid in England, used by around 6% of adults.^1^ While the long-term health consequences remain unknown, vaping is now widely considered to be markedly less harmful than smoking.^5 6^ Some research suggests that vaping may be associated with increased rates of smoking cessation,^17^,^9^ and may be a more effective cessation aid than nicotine replacement therapy.^10^ However, recent meta-analysis suggests that while e-cigarette provision as a therapeutic intervention was associated with increased smoking cessation in randomised controlled trials, e-cigarette use as a consumer product was not associated with smoking cessation in observational studies.^11^ Moreover, current evidence suggests that, among ex-smokers, vaping may increase smoking relapse risks.^12 13^ The frequency of e-cigarette use and the type of device used is also consequential, as some research suggests that those vaping less frequently and/or using less advanced devices are less likely to quit smoking/more likely to relapse.^9 14^

One important aspect of e-cigarette usage relates to its impacts on socioeconomic inequalities. Smoking cessation has tended to be less likely for smokers in a more disadvantaged socioeconomic position (SEP), with disadvantaged smokers being less likely to quit/more likely to relapse, but not less likely to want to quit.^15^,^19^ Theoretically, e-cigarettes may potentially reduce this socioeconomic inequality if they can make smoking cessation more accessible for disadvantaged smokers, but conversely may widen inequalities if vaping exposes disadvantaged ex-smokers to increased relapse risk.^20 21^ Importantly, inequalities in smoking cessation have narrowed recently since e-cigarettes have become more widely available,^22^ though it is not clear whether this occurred because of increased vaping or other confounding factors.

Current evidence on the impact of vaping on socioeconomic inequalities in smoking cessation/relapse is fairly limited. One review suggests e-cigarette ‘awareness’, ‘ever use’ and ‘current use’ are patterned by a range of sociodemographic factors, but that overall there is a lack of a clear pattern in these outcomes with regard to SEP, particularly in high-quality studies.^23^ US data suggest that socioeconomic inequalities in smoking cessation remained unchanged from 2014 to 2019 and that attempts to quit via vaping were higher among those in higher SEP groups.^24^ Conversely, data from England suggest that e-cigarette use increased for all SEP groups from 2014 to 2019 but was highest among those from lower SEP groups.^21^ Finally, UK cross-sectional research suggests that socioeconomic inequalities in smoking cessation were weaker among those who vaped.^20^ This highlights that, while more research is needed, e-cigarettes may potentially narrow health inequalities by helping disadvantaged smokers to quit, and suggests that vaping may have contributed to the recent reduction in inequalities in smoking cessation in the UK.^22^

The interplay between vaping and smoking can be complex, involving, for example, patterns of dual use (with or without intentions to quit smoking), switching fully from smoking to vaping or using vaping as a ‘stepping stone’ to stop smoking and eventually cease nicotine use.^125^,^27^ However, since smoking is considered far more harmful than vaping,^5 6^ inequalities in smoking are of more critical public health importance. With the potential both for inequalities in vaping behaviour and for effects of vaping on cessation and relapse rates it may be helpful to frame the issue in terms of whether vaping mediates or moderates inequalities in smoking cessation/relapse. Importantly, ‘mediation’ could include ‘suppression’ effects,^28^ where, for example, vaping might be more common among disadvantaged smokers and might help them quit, thus leading to narrower inequalities in cessation than would be present without access to e-cigarettes. Even without inequalities in vaping, it is possible that vaping could impact inequalities in smoking if it moderates associations between SEP and cessation/relapse.^29^

The aim of this study is to assess whether vaping mediates or moderates socioeconomic inequalities in smoking cessation/relapse. Specifically, the following research questions (RQ) are addressed over 2 years of follow-up:

RQ1: Among current smokers:

- Is SEP associated with vaping?
- Is vaping associated with smoking cessation?
- Is SEP associated with smoking cessation?
- Does vaping mediate or moderate associations between SEP and smoking cessation?

RQ2: Among ex-smokers:

- Is SEP associated with vaping?
- Is vaping associated with smoking relapse?
- Is SEP associated with smoking relapse?
- Does vaping mediate or moderate associations between SEP and smoking relapse?

## Methods

### Data and sample

Analyses used longitudinal data from waves 8–10 of the UK Household Longitudinal Study (UKHLS), a nationally representative household panel study based on a clustered-stratified probability sample of ~40 000 UK households.^30^ UKHLS data collection began in 2009–2011, and individuals from the same households are interviewed annually face-to-face or online. Our analysis primarily used wave 8 (2016–2018), wave 9 (2017–2019) and wave 10 (2018–2020) data, although some information from earlier waves was used where applicable (see below). Waves 8–10 were selected as they included more detailed categorisations of vaping status than previous waves, and are the most recent waves which were unaffected by the COVID-19 pandemic. Details of UKHLS response rates are available online.^31^

Using smoking status at wave 8 as a baseline, smoking cessation/relapse was then measured over the following 2 years (waves 9–10). UKHLS respondents were included in our analysis if they met the following inclusion criteria: (1) were interviewed at wave 8, (2) had a valid, non-missing wave 8 weight and (3) had data on smoking status at wave 9 or 10. This gave a final primary sample of 25 102 individuals (see online supplemental appendix A for details of sample size/exclusions/missing data). All analyses were conducted using Stata/MP V.17.0. Wave 8 weights were applied to adjust for survey design/non-response, and we applied additional weighting using baseline variables for having smoking data at waves 9 and 10. Item non-response was dealt with via multiple imputation, using chained equations,^32^ with 50 imputations added (see online supplemental appendix A for details of missingness across variables).

### Measured variables

Our sample was stratified by baseline (wave 8) smoking status (1=never smoker, 2=ex-smoker, 3=current smoker). Respondents were categorised as current smokers if they self-reported being a smoker at wave 8. Those who self-reported being a smoker in earlier wave(s), or historic daily smoking, were categorised as ex-smokers. Remaining respondents were categorised as never smokers. Outcomes were binary indicators measuring: (1) smoking cessation by wave 9 or 10 among wave 8 smokers (0=no, 1=yes), and (2) smoking relapse by wave 9 or 10 among wave 8 ex-smokers (0=no, 1=yes). Our main exposure variable, SEP, was represented using educational attainment (0=degree, including higher degree/first degree or equivalent/diploma in higher education/teaching or nursing qualification, 1=no degree). Wave 8 self-reported regular (ie, at least weekly) vaping status was defined as a mediator (0=not regular vaper, 1=regular vaper).

**Figure 1:**
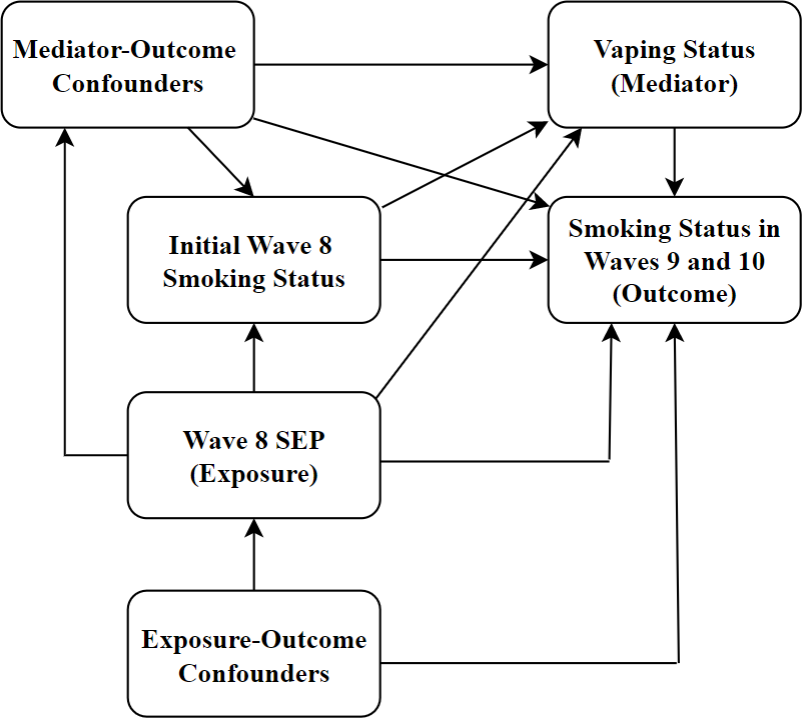
Causal diagram of the relationship between SEP, vaping and smoking cessation/relapse.

Causal relationships between SEP, vaping and smoking cessation/relapse are complex, with various potential confounders at different stages of the causal pathway (see figure 1). Consequently, our analysis included a list of: (1) exposure-outcome (and exposure-mediator) confounders, that is, potential determinants of both exposure (wave 8 SEP), mediator (wave 8 vaping) and outcome (smoking cessation/relapse at wave 9 or 10); and (2) mediator-outcome confounders, that is, potential determinants of both mediator (wave 8 vaping) and outcome (smoking cessation/relapse at wave 9 or 10), some of which may have been caused by the exposure (wave 8 SEP). Since these groups of variables have different roles in the causal pathway they were treated differently in our analysis (see the statistical analysis section).

Exposure-outcome variables were: sex (0=male, 1=female), age group (1=16–24, 2=25–34, 3=35–44, 4=45–54, 5=55+), UK country (1=England, 2=Wales, 3=Scotland, 4=Northern Ireland), ethnicity (0=white, 1=non-white) and rurality (0=rural, 1=urban). Mediator-outcome variables were: partner status (0=in couple, 1=single), has kids (0=no, 1=yes), housing tenure (0=owner, 1=renter), National Statistics Socio-economic Classification (NSSEC) (1=management/professional, 2=intermediate, 3=routine, 4=not in paid employment), has long-standing illness (0=no, 1=yes), vaping history (0=does not vape at all at wave 7, 1=vapes at all at wave 7), mental health (measured by General Health Questionnaire (GHQ)) (1=GHQ <4, 2=GHQ 4+), poverty status (0=not in poverty, 1=in poverty), age started smoking (0=0–15, 1=16–18, 2=19–25, 3=>25) and smoking history, that is, mean number of cigarettes per day across waves or when last smoked regularly (0=0–10, 1=11–20, 2=>20). With the exception of the vaping and smoking history variables, all exposure-outcome/mediator-outcome variables were measured at wave 8 (or wave 7 if wave 8 data were missing).

### Statistical analysis

Our analysis plan was preregistered using Open Science Framework (available: https://osf.io/e3z8q). Our reporting is consistent with the Strengthening the Reporting of Observational Studies in Epidemiology guidelines (see online supplemental appendix B). First, we used logistic regression to estimate unadjusted associations between the variables of interest in each RQ. These unadjusted associations may be subject to collider bias,^33 34^ because the data are stratified by wave 8 smoking status, which is potentially determined by both (1) the exposure variable and (2) other variables determining cessation/relapse. This is shown in figure 1.

Second, to account for this, we used inverse probability weighted marginal structural models to estimate controlled direct effects (CDE) of SEP on smoking cessation/relapse, controlling for observed confounding, including mediator-outcome confounders that are affected by the exposure.^35^ The CDE represents the effect of the exposure, with mediators set to a particular level (eg, setting wave 8 status to either current smoking or ex-smoking, and to either regular vaping or not regular vaping). Weights were calculated within each imputed data set and final results were aggregated across imputed data sets using Rubin’s rules.^32^ These models aim to remove any imbalance of observed confounders across exposure levels that is not caused by the exposure. CDE estimates account for interactions between the exposure and the mediators and may therefore vary depending on the values mediators are set to.^35^ As explained below, some of our CDE estimates treat wave 8 smoking status as the only mediator, so provide estimates with wave 8 smoking set to either current smoking or ex-smoking (to get separate estimates for cessation and relapse). Later estimates include vaping as an additional mediator and compare estimates with vaping set to regular or not regular vaping. We estimate effects across two waves of follow-up using a discrete-time, event history approach, with up to two rows of data for waves 9 and 10; the wave 10 row is censored if cessation/relapse occurs at wave 9. Thus, ORs can be interpreted as the hazard or risk of cessation/relapse in a given year if this has not already occurred.

For part (a) of our RQs (Is SEP associated with vaping?), we created a weight to estimate the CDE of education with wave 8 smoking status set to either current smoking or ex-smoking. This adjusts for (exposure-outcome) confounders of education, vaping and smoking through follow-up, and for (mediator-outcome) confounders of wave 8 smoking status, vaping and smoking through follow-up. A similar set of weights were then used for part (b) of our RQs (Is vaping associated with smoking cessation/relapse?), but with vaping treated as the exposure rather than education, and cessation/relapse as the outcome. For part (c) of our RQs (Is SEP associated with smoking cessation/relapse?) the same weights as part (a) were used to estimate the CDE of education on smoking cessation/relapse. Finally, for part (d) of our RQs (Does vaping mediate or moderate associations between SEP and smoking cessation/relapse?), the same inverse probability weights used for parts (a) and (b) were used, but with an additional step of weighting to account for regular vaping as the mediator. We produced separate CDE estimates for effects of education on cessation/relapse with vaping status set to either regular or not regular vaping. For full details of the process of creating the weights and running the modelling for each RQ, see online supplemental appendix C.

Finally, we conducted additional sensitivity analyses. First, vaping status was recoded to indicate any vaping (0=non-vaper, 1=infrequent/regular vaper). Next, we used two binary classifications of NSSEC as our main SEP measure (0=management/professional, 1=not management/professional; and 0=in paid employment, 1=not in paid employment), with education reclassified as an exposure-outcome confounder. This assesses whether there is evidence for any additional effect of a more proximal SEP measure, over and above the effect of the education measure used in the main analyses. Lastly, analyses were repeated with education recoded to indicate possession of any qualifications (0=has qualifications, including degree or any school-level qualifications, 1=no qualifications).

## Results

### Descriptive statistics

Descriptive statistics showing sociodemographic patterning of our sample by wave 8 smoking and vaping status are provided in table 1. Overall, 16.1% were smokers and 30.1% ex-smokers. Smoking was disproportionately prevalent among people without degrees, as well as among those who were single, renting, younger, in urban areas, in poverty, or with a long-standing illness or higher GHQ scores. Regular vaping was rare overall (4.0% of sample), but was more prevalent among smokers (8.8%) and ex-smokers (8.4%). Vaping was also disproportionately prevalent among those without degrees and those who were male, aged 25–34, white, renting, in urban areas or with kids in their household. In addition to table 1, online supplemental appendix D table S2 also provides descriptive statistics showing how smoking cessation/relapse outcomes vary by SEP and vaping status.

**Table 1:** Sociodemographic patterning of sample by wave 8 smoking and vaping status

	Unweighted n (weighted %)					
	Total	Smoking status			Vaping status	
Covariates		Never smokers	Ex-smokers	Current smokers	Non-regular vapers	Regular vapers
	25 102 (100)	13 511 (53.7)	7724 (30.1)	3867 (16.1)		
Smoking status						
Never smoker					13 489 (55.9)	22 (2.5)
Ex-smoker					7134 (28.8)	590 (62.3)
Current smoker					3513 (15.3)	354 (35.2)
Vaping status						
Non-regular vaper	24 136 (96.0)	13 489 (99.8)	7134 (91.6)	3513 (91.2)		
Regular vaper	966 (4.0)	22 (0.2)	590 (8.4)	354 (8.8)		
Degree						
No degree	15 743 (63.8)	7603 (57.3)	5090 (66.6)	3049 (80.4)	15 036 (63.3)	706 (75.0)
Has degree	9359 (36.2)	5907 (42.7)	2634 (33.4)	818 (19.6)	9100 (36.7)	259 (25.0)
Sex						
Male	11 035 (47.8)	5394 (45.3)	3800 (51.1)	1841 (49.7)	10 544 (47.5)	491 (54.1)
Female	14 067 (52.2)	8117 (54.7)	3924 (48.9)	2026 (50.3)	13 592 (52.5)	475 (45.9)
Age						
16–24	1601 (12.8)	1062 (16.4)	153 (4.2)	386 (17.1)	1543 (13.0)	58 (9.7)
25–34	2682 (13.1)	1520 (13.6)	561 (9.3)	601 (18.4)	2524 (12.7)	158 (20.9)
35–44	4028 (14.9)	2235 (14.8)	1083 (14.3)	710 (16.5)	3821 (14.7)	207 (19.6)
45–54	5037 (17.9)	2780 (17.7)	1427 (17.7)	830 (18.7)	4804 (17.7)	233 (21.9)
55+	11 754 (41.3)	5914 (37.5)	4500 (54.6)	1340 (29.3)	11 444 (41.9)	310 (27.9)
Ethnicity						
White	22 087 (92.0)	11 468 (89.3)	7230 (95.6)	3389 (94.2)	21 199 (91.9)	888 (95.3)
Non-white	3015 (8.0)	2043 (10.7)	494 (4.4)	478 (5.8)	2937 (8.1)	78 (4.7)
NSSEC						
Management/professional	6426 (24.7)	4066 (29.4)	1748 (22.2)	612 (13.8)	6207 (24.8)	219 (21.5)
Intermediate	3516 (13.7)	2016 (15.0)	985 (12.1)	515 (12.1)	3348 (13.6)	168 (15.8)
Routine	4569 (20.3)	2206 (18.6)	1257 (17.9)	1106 (30.4)	4313 (20.0)	256 (28.6)
Not in paid employment	10 591 (41.3)	5223 (36.9)	3734 (47.8)	1634 (43.7)	10 268 (41.6)	323 (34.0)
Single in household						
No	16 834 (59.7)	9248 (59.1)	5499 (67.5)	2087 (46.8)	16 218 (59.7)	616 (57.8)
Yes	8268 (40.3)	4263 (40.9)	2225 (32.5)	1780 (53.2)	7918 (40.3)	350 (42.2)
Kids in household						
No	18 681 (76.2)	9899 (76.8)	5975 (76.7)	2807 (72.9)	18 012 (76.4)	669 (69.8)
Yes	6421 (23.8)	3612 (23.2)	1749 (23.3)	1060 (27.1)	6124 (23.6)	297 (30.2)
Tenure						
Owner	18 960 (67.9)	11 057 (75.0)	5940 (69.4)	1963 (41.5)	18 381 (68.7)	579 (49.1)
Renter	6142 (32.1)	2454 (25.0)	1784 (30.6)	1904 (58.5)	5755 (31.3)	387 (50.9)
Rural/urban						
Rural	6678 (24.2)	3624 (24.4)	2224 (25.8)	830 (20.5)	6477 (24.4)	201 (19.8)
Urban	18 424 (75.8)	9887 (75.6)	5500 (74.2)	3037 (79.5)	17 659 (75.6)	765 (80.2)
Has long-standing illness						
No	15 596 (63.1)	9028 (68.3)	4293 (55.5)	2275 (59.8)	15 013 (63.3)	583 (59.4)
Yes	9506 (36.9)	4483 (31.7)	3432 (44.5)	1592 (40.2)	9123 (36.7)	383 (40.6)
In poverty						
No	21 951 (87.2)	11 953 (88.7)	6845 (88.0)	3153 (80.6)	21 105 (87.3)	846 (85.7)
Yes	3151 (12.8)	1558 (11.3)	879 (12.0)	714 (19.4)	3031 (12.7)	120 (14.3)
GHQ						
<4 (less distressed)	20 519 (80.7)	11 278 (83.0)	6356 (80.8)	2885 (73.1)	19 759 (80.9)	760 (77.4)
4+ (more distressed)	4583 (19.3)	2233 (17.0)	1368 (19.2)	981 (26.9)	4377 (19.1)	206 (22.6)
Wave 7 e-cigarettes ever use						
No	23 331 (92.6)	13 423 (99.3)	7085 (91.0)	2823 (73.1)	23 033 (95.2)	298 (31.4)
Yes	1771 (7.4)	88 (0.7)	639 (9.0)	1044 (26.9)	1103 (4.8)	668 (68.6)
Current/ex-smoker mean number of cigarettes per day across waves						
<11	5535 (47.9)		3393 (44.2)	2142 (54.8)	5130 (48.5)	405 (41.7)
11–20	4539 (39.2)		3074 (39.7)	1465 (38.2)	4142 (38.8)	398 (43.3)
>20	1517 (12.9)		1257 (16.1)	260 (7.0)	1376 (12.7)	141 (15.0)
Current/ex-smoker age started smoking						
<16	4552 (41.6)		2782 (37.3)	1770 (49.6)	4131 (41.1)	420 (46.6)
16–19	5010 (42.7)		3495 (45.0)	1515 (38.4)	4627 (43.0)	383 (39.4)
19–25	1568 (12.3)		1127 (13.8)	441 (9.4)	1455 (12.3)	113 (11.5)
>25	461 (3.5)		320 (3.9)	141 (2.7)	433 (3.6)	28 (2.6)

Notes: Multiple imputed data with 50 imputations added. Weighting uses UK Household Longitudinal Study (UKHLS) wave 8 sampling weight.

GHQGeneral Health QuestionnaireNSSECNational Statistics Socio-economic Classification

### Effects of SEP on regular vaping

Table 2 shows the estimated effects of education on vaping among wave 8 current smokers and ex-smokers. Both unadjusted and adjusted CDE estimates are provided. Among current smokers, having no degree was associated with regular vaping, but CIs overlapped the null in both the unadjusted (OR: 1.28; 95% CI 0.93–1.76) and adjusted (OR: 1.24; 95% CI 0.87–1.78) models. Among ex-smokers, having no degree was associated with increased odds of regular vaping in both unadjusted (OR: 1.27; 95% CI 1.02–1.60) and adjusted (OR: 1.66; 95% CI 1.33–2.07) models.

**Table 2:** Estimated effects of SEP on regular vaping among current smokers and ex-smokers

	Unadjusted association between having no degree and regular vaping	Controlled direct effect of having no degree on regular vaping
	OR (95% CI)	OR (95% CI)
Wave 8 regular vaping (current smokers)		
(Reference: Degree)		
No degree	1.28 (0.93–1.76)	1.24 (0.87–1.78)
Wave 8 regular vaping (ex-smokers)		
(Reference: Degree)		
No degree	1.27 (1.02–1.60)	1.66 (1.33–2.07)

Notes: ratio and CI confidence interval. Regular vaping is defined as vaping at least weekly. The unadjusted association uses UK Household Longitudinal Study (UKHLS) wave 8 sampling weights to account for survey design and non-response but does not adjust for any confounders. The controlled direct effect uses inverse probability weighted marginal structural modelling to additionally adjust for exposure-outcome confounders and mediator-outcome confounders.

SEPsocioeconomic position

### Effects of regular vaping on smoking cessation/relapse

Table 3 shows the estimated effects of regular vaping on smoking cessation/relapse, again providing both unadjusted and adjusted CDE estimates. Regular vaping was associated with increased odds of smoking cessation among wave 8 current smokers (OR: 1.28; 95% CI 1.03–1.59), but this was attenuated after adjusting for observed confounding (OR: 1.13; 95% CI 0.82–1.55). Among wave 8 ex-smokers, regular vaping was associated with increased odds of smoking relapse in both unadjusted (OR: 2.75; 95% CI 2.02, 3.73) and adjusted (OR: 2.97; 95% CI 2.10–4.22) models.

**Table 3:** Estimated effects of regular vaping on smoking cessation/relapse

	Unadjusted association between wave 8 regular vaping and smoking cessation/relapse	Controlled direct effect of wave 8 regular vaping on smoking cessation/relapse
	OR (95% CI)	OR (95% CI)
Smoking cessation		
(Reference: Not regular vaper)		
Regular vaper	1.28 (1.03–1.59)	1.13 (0.82–1.55)
Smoking relapse		
(Reference: Not regular vaper)		
Regular vaper	2.75 (2.02–3.73)	2.97 (2.10–4.22)

Notes: ratio and CI confidence interval. Regular vaping is defined as vaping at least weekly. The unadjusted association uses UK Household Longitudinal Study (UKHLS) wave 8 sampling weights to account for survey design and non-response but does not adjust for any confounders. The controlled direct effect uses inverse probability weighted marginal structural modelling to additionally adjust for exposure-outcome confounders and mediator-outcome confounders.

### Effects of SEP, and its interaction with regular vaping, on smoking cessation/relapse

Table 4 shows the relationship between SEP and smoking cessation/relapse with unadjusted associations, CDE estimates adjusting for confounding but not for vaping and CDE estimates dependent on regular vaping status. If vaping mediates inequalities in smoking cessation/relapse, then the estimates dependent on regular vaping status (columns 3 and 4) would be reduced relative to associations not conditioned on vaping (column 2). If vaping moderates inequalities in smoking cessation/relapse, then the estimates dependent on regular vaping status will differ from each other. Among wave 8 current smokers, having no degree was associated with reduced odds of smoking cessation. This was consistent across unadjusted (OR: 0.62; 95% CI 0.52–0.73) and confounder-adjusted models (OR: 0.65; 95% CI 0.54–0.77). A similar relationship was present among those who were not regular vapers (OR: 0.62; 95% CI 0.50–0.76), but the association disappeared for regular vapers (OR: 0.99; 95% CI 0.54–1.82).

**Table 4:** Estimated effects of SEP on smoking cessation/relapse with and without interaction by regular vaping status

	Unadjusted association between having no degree and smoking cessation/relapse	Controlled direct effect of having no degree on smoking cessation/relapse	Controlled direct effect of having no degree on smoking cessation/relapseamong non-regular vapers	Controlled direct effect of having no degree on smoking cessation/relapseamong regular vapers
	OR (95% CI)	OR (95% CI)	OR (95% CI)	OR (95% CI)
Smoking cessation				
(Reference: Degree)				
No degree	0.62 (0.52–0.73)	0.65 (0.54–0.77)	0.62 (0.50–0.76)	0.99 (0.54–1.82)
Smoking relapse				
(Reference: Degree)				
No degree	1.34 (1.04–1.72)	1.74 (1.37–2.22)	1.55 (1.09–2.18)	2.13 (1.05–4.29)

Notes: ratio and CI confidence interval. Regular vaping is defined as vaping at least weekly. The unadjusted association uses UK Household Longitudinal Study (UKHLS) wave 8 sampling weights to account for survey design and non-response but does not adjust for any confounders. The controlled direct effect uses inverse probability weighted marginal structural modelling to additionally adjust for exposure-outcome confounders and mediator-outcome confounders.

SEPsocioeconomic position

Among wave 8 ex-smokers, having no degree was associated with raised risk of relapse in unadjusted (OR=1.34; 95% CI 1.04–1.72) and confounder-adjusted (OR: 1.74; 95% CI 1.37–2.22) models. After regular vaping was included, the association remained present among regular vapers (OR: 2.13; 95% CI 1.05–4.29) and those who were not regular vapers (OR: 1.55; 95% CI 1.09–2.18).

### Sensitivity analysis

Findings from sensitivity analyses in which vaping status was recoded to include infrequent vapers were broadly consistent with the main analysis (see online supplemental appendix E). Analyses using NSSEC suggested little remaining socioeconomic inequality in cessation/relapse after adjusting for educational attainment (see online supplemental appendices F and G). Nevertheless, despite wide CIs, both analyses showed cessation as being less likely in disadvantaged occupations, with a similar association for those who did not regularly vape, while for regular vapers the association had reversed in direction. One other difference worth noting is that respondents not in employment had lower odds of vaping among both current smokers and ex-smokers than those in employment.

Recoding our education measure to indicate no qualifications produced notably different findings (see online supplemental appendix H). Respondents with no qualifications were less likely to be regular vapers (unadjusted OR: 0.69; 95% CI 0.54–0.87; CDE OR: 0.86; 95% CI 0.67–1.11) than those with qualifications. Moreover, while smoking cessation was less likely among those with no qualifications this association was present among regular vapers (OR: 0.30; 95% CI 0.14–0.65) and those who were not regular vapers (OR: 0.75; 95% CI 0.56–0.99). Together with our main analyses, this suggests a non-linear relationship, whereby vaping may help reduce socioeconomic inequalities in smoking cessation at the middle/upper end of the educational distribution (ie, between those with/without degrees), but is unlikely to help reduce inequalities at the lower end of the educational distribution (ie, between those with/without any qualifications).

## Discussion

This study has examined the impact of vaping on socioeconomic inequalities in smoking cessation/relapse using UKHLS data spanning 2016 to early 2020. Our findings suggest that smokers with lower educational attainment were less likely to stop smoking, but this inequality was not present among smokers who vaped regularly. However, vaping only appeared to alleviate inequalities when comparing those at the top of the educational distribution (those with degrees) to those in the middle/bottom (those without degrees). It did not appear to alleviate inequalities at the lower end of the distribution, between those with no qualifications and those who did have some. With regard to smoking relapse, our findings suggest that ex-smokers with less education were more likely to relapse, SEP was associated with vaping among ex-smokers and vaping was associated with relapse. These relationships did not appear strong enough for our final analysis to show clear evidence of mediation or moderation of inequalities in relapse by vaping status.

Importantly, if e-cigarettes can be particularly useful in helping disadvantaged groups to quit smoking, then this could lead to long-term reductions in health inequalities. Overall, data from England suggest that socioeconomic inequalities in cessation have narrowed recently.^22^ Our findings suggest that increased vaping among those of lower SEP (ie, without degrees) is likely to have contributed positively to this, as smoking cessation is less strongly associated with having degree level education among regular vapers. We confirm previous cross-sectional research where inequalities were found to be weaker among adult vapers,^20^ but our study extends this finding with longitudinal data. We also demonstrate that the impact of vaping on inequalities is focused around the upper/middle end of the educational distribution, but does little to help those who are most disadvantaged, or to address inequalities in relapse among ex-smokers.

Our study has some limitations. First, while we adjust for many relevant confounders, causal interpretation is based on assumptions of no unmeasured confounding. Since our analysis was stratified by wave 8 smoking status, this includes unmeasured confounding of smoking at wave 8 and through follow-up in waves 9 and 10 (ie, any unmeasured determinant of continued smoking). One obvious candidate for an unmeasured confounder is residual differences in smoking history, which we did adjust for, but the measures were crude (being based on limited data from earlier surveys) and may not fully reflect smoking history differences between smoking/vaping categories. It is plausible that bias arising from this, for example, may have contributed to the observed association between vaping and greater risk of smoking relapse. An additional limitation is that our smoking cessation measure is based on self-reported smoking status between waves, and we do not know how long respondents had quit for. Finally, UKHLS data do not distinguish between different device types or different motivations for vaping.

Despite these limitations, our findings have some important implications. While inequalities in smoking cessation have previously been intractable, our findings highlight that vaping may help alleviate inequalities between those with/without degrees. This suggests that e-cigarette policy/regulations should consider that vaping may be especially helpful as a cessation aid for smokers without degree level education and therefore may help reduce inequalities in smoking. Concerns remain because the long-term health consequences of vaping are unknown and some fear potential ‘gateway effects’ between vaping and smoking uptake. However, vaping is now widely considered to be substantially less harmful than smoking,^5 6^ and latest evidence suggests ‘gateway effects’ are unlikely.^36^ Our findings did not show that vaping helped with inequalities between those with/without any qualifications, or with inequalities in smoking relapse, although there was not clear evidence of an adverse impact on inequalities in relapse either. Therefore, other cessation aids may be more useful to those most disadvantaged (ie, with no qualifications), and may be needed for avoiding relapse. Nonetheless, a reduction in inequalities in smoking cessation is significant and likely means that vaping can have a net positive impact on inequalities in smoking.

## supplementary material

10.1136/tc-2022-057728 online supplemental file 1

## Data Availability

Data may be obtained from a third party and are not publicly available.
