# Supplementary material for: Vaping and socioeconomic inequalities in smoking cessation and relapse: a longitudinal analysis of the UK Household Longitudinal Study
Source: Tob Control. 2023 Apr 11;33(e2):e057728. doi: 10.1136/tc-2022-057728 (PMC11672026; doi:10.1136/tc-2022-057728)
Supplement: online supplemental file 1 [file tc-33-e2-s001.pdf]

SUPPORTING INFORMATION APPENDIX A

Figure S1. Flowchart showing sample size and missing data at each stage of inclusion criteria

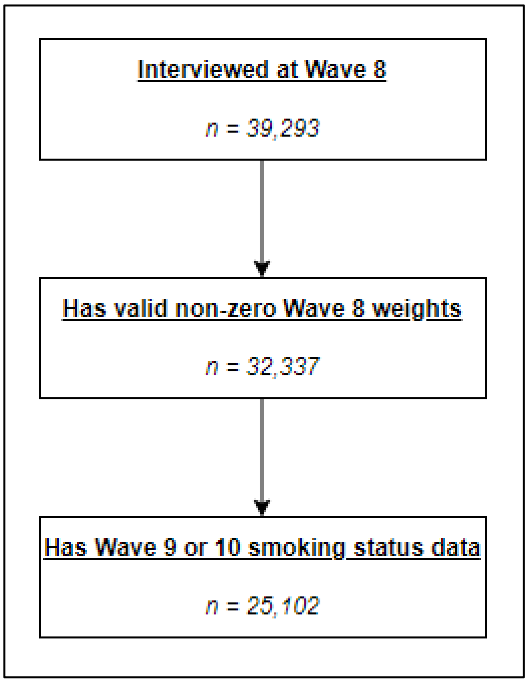

Table S1. Missingness Across Covariates in Final Primary Sample

	Missing N (Weighted % of Observations)			
**Covariate**	**Total**	**Never Smokers**	**Ex-Smokers**	**Current Smokers**
Degree	3,656 (10.8%)	1,867 (10.1%)	1,224 (12.6%)	565 (9.7%)
GHQ	871 (2.8%)	505 (3.0%)	222 (2.34%)	144 (3.0%)
Vaping History: wave 7 E-Cigs Ever Use	579 (2.9%)	285 (2.9%)	100 (1.5%)	194 (5.5%)
Smoking History: current/ex-smoker mean # cigarettes per day across waves	1,243 (5.3%)	0	1,168 (15.1%)	75 (2.0%)
Smoking History: current/ex-smoker age started smoking	824 (3.0%)	0	175 (2.6%)	649 (13.9%)

*Note: all other covariates (sex, age, ethnicity, NSSEC, single in household, kids in household, tenure, rural/urban, longstanding illness and poverty) had full data in primary sample.

SUPPORTING INFORMATION APPENDIX B

STROBE Checklist of items that should be included in reports of observational studies

	Item	Recomendation
**Title and abstract**	1	(a) Indicate the study's design with a commonly used term in the title or the abstract<br>(b) Provide in the abstract an informative and balanced summary of what was done and what was found
**Introduction**		
Background/rationale	2	Explain the scientific background and rationale for the investigation being reported
Objectives	3	State specific objectives, including any prespecified hypotheses
**Methods**		
Study design	4	Present key elements of study design early in the paper
Setting	5	Describe the setting, locations, and relevant dates, including periods of recruitment, exposure, follow-up, and data collection
Participants	6	(a) Cohort study—Give the eligibility criteria, and the sources and methods of selection of participants. Describe methods of follow-up Case-control study—Give the eligibility criteria, and the sources and methods of case ascertainment and control selection. Give the rationale for the choice of cases and controls Cross-sectional study—Give the eligibility criteria, and the sources and methods of selection of participants<br>(b) Cohort study—For matched studies, give matching criteria and number of exposed and unexposed Case-control study—For matched studies, give matching criteria and the number of controls per case
Variables	7	Clearly define all outcomes, exposures, predictors, potential confounders, and effect modifiers. Give diagnostic criteria, if applicable
Data sources/ measurement	8	For each variable of interest, give sources of data and details of methods of assessment (measurement). Describe comparability of assessment methods if there is more than one group
Bias	9	Describe any efforts to address potential sources of bias
Study size	10	Explain how the study size was arrived at

Quantitative variables	11	Explain how quantitative variables were handled in the analyses. If applicable, describe which groupings were chosen and why
Statistical methods	12	(a) Describe all statistical methods, including those used to control for confounding<br>(b) Describe any methods used to examine subgroups and interactions<br>(c) Explain how missing data were addressed<br>(d) Cohort study—If applicable, explain how loss to follow-up was addressed Case-control study—If applicable, explain how matching of cases and controls was addressed Cross-sectional study—If applicable, describe analytical methods taking account of sampling strategy<br>(e) Describe any sensitivity analyses
**Results**		
Participants	13	(a) Report numbers of individuals at each stage of study—eg numbers potentially eligible, examined for eligibility, confirmed eligible, included in the study, completing follow-up, and analysed<br>(b) Give reasons for non-participation at each stage<br>(c) Consider use of a flow diagram
Descriptive data	14	(a) Give characteristics of study participants (eg demographic, clinical, social) and information on exposures and potential confounders<br>(b) Indicate number of participants with missing data for each variable of interest<br>(c) Cohort study—Summarise follow-up time (eg, average and total amount)
Outcome data	15	Cohort study—Report numbers of outcome events or summary measures over time<br>Case-control study—Report numbers in each exposure category, or summary measures of exposure<br>Cross-sectional study—Report numbers of outcome events or summary measures
Main results	16	(a) Give unadjusted estimates and, if applicable, confounder-adjusted estimates and their precision (eg, 95% confidence interval). Make clear which confounders were adjusted for and why they were included<br>(b) Report category boundaries when continuous variables were categorized<br>(c) If relevant, consider translating estimates of relative risk into absolute risk for a meaningful time period
Other analyses	17	Report other analyses done—eg analyses of subgroups and interactions, and sensitivity analyses

**Discussion**		
Key results	18	Summarise key results with reference to study objectives
Limitations	19	Discuss limitations of the study, taking into account sources of potential bias or imprecision. Discuss both direction and magnitude of any potential bias
	20	Give a cautious overall interpretation of results considering objectives, limitations, multiplicity of analyses, results from similar studies, and other relevant evidence
Generalisability	21	Discuss the generalisability (external validity) of the study results
**Other information**		
Funding	22	Give the source of funding and the role of the funders for the present study and, if applicable, for the original study on which the present article is based

SUPPORTING INFORMATION APPENDIX C

FULL DETAILS OF STATISTICAL ANALYSIS BY RESEARCH QUESTION

Research Questions part (a): Is SEP associated with vaping?

The primary sample with multiple imputations was used to create a stabilised inverse probability weight for the exposure (E) using our set of exposure-outcome confounders (x). This was based on the following two logistic regression models: (1) SEP as outcome, with no predictors (i.e. estimating: P(E)), and (2) SEP as outcome, with all exposure-outcome confounders as predictors (i.e. estimating: P(E|x)). This was used to calculate the predicted probabilities of each respondents' observed exposure level (P*(E) and P*(E|x), where P*=P if the exposure=1 and P*=1-P if the exposure=0). Then, the exposure weight was calculated as P*(E)/P*(E|x).

Next, we created an inverse probability weight for w8 smoking status (M), conditioned on both exposure-outcome confounders (x), and mediator outcome confounders (z), using the following two logistic regression models: (1) wave 8 smoking status as outcome, predicted only by SEP (i.e. estimating: P(M|E)), and (2) wave 8 smoking status as outcome, predicted by SEP, and all exposure-outcome and mediator-outcome confounders (i.e. estimating: P(M|E, x, z)). This was conducted in two stages, with the first stage being conducted for ever vs. never smokers and the second stage for ever vs. current smokers. The vaping and smoking history variables were only included in the second stage as they were either highly correlated or deterministically-related to smoking status. This was used to calculate predicted probabilities (P*) of each respondents' observed wave 8 smoking status, and the wave 8 smoking status weight is calculated as P*(M|E)/P*(M|E, x, z). This was multiplied together with the exposure weight (and the sampling/non-response weights) and represents respondents' wave 9 analysis weight. Finally, a further weight was created for having smoking data at wave 10 (C), conditional on wave 9 smoking status (Y9). This was based on the following models: (1) Smoking data at wave 10 as outcome, predicted only by SEP, w8 smoking status and w9 smoking status (i.e. estimating: P(C|E, M, Y9)), and (2) Smoking data at wave 10 as outcome, predicted by SEP, w8 smoking status, w9 smoking status and all confounders (i.e. estimating: P(C|E, M, Y9, x, z)). An inverse probability weight was created using P* as above, and this is multiplied together with the wave 9 analysis weight to create a wave 10 analysis weight.

Logistic regression models were then used, with this final analysis weight applied, to examine the relationship between education and vaping, with wave 8 smoking status set to either current or ex-smoking. The odds ratio from these models can be interpreted as the CDE of education on vaping.

Research Questions part (b): Is vaping associated with smoking cessation?

In a similar process to research question 1, we created two inverse probability weights based on the following two logistic regression models: (1) wave 8 smoking status as outcome, predicted only by regular vaping status, and (2) wave 8 smoking status as outcome, predicted by regular vaping status and all exposure-outcome and mediator-outcome confounders. These weights were then multiplied together and used in logistic regression models, which estimate the CDE of regular vaping on smoking cessation/relapse.

Research Questions part (c): Is SEP associated with smoking cessation/relapse?

The same weights as research question 1 were used to estimate the CDE of education on smoking cessation/relapse.

Research Questions part (d): Does vaping mediate or moderate the associations between SEP and smoking cessation/relapse?

The same inverse probability weights used for research questions 1 and 2 were used, but with an additional step of weighting for regular vaping as the mediator. These weights were multiplied together and used in logistic regression models with smoking cessation/relapse as the outcome and SEP, wave 8 regular vaping, and the interaction between the two included as predictors. This estimates the CDE of education on smoking cessation/relapse with vaping status set to either regular vaping or not regular vaping.

SUPPORTING INFORMATION APPENDIX D

Table S2. Patterning of Smoking Cessation Outcomes (Among Wave 8 Current Smokers) and Smoking Relapse Outcomes (Among Wave 8 Ex-Smokers) by Vaping Status and Socioeconomic Position (Measured by Having Degree Level Education)

	Unweighted N (Weighted Row %)							
	**Smoking Cessation** (Among Wave 8 Current Smokers, N = 3,867)				**Smoking Relapse** (Among Wave 8 Ex-Smokers, N = 7,724)			
	**By Wave 9**		**By Wave 10**		**By Wave 9**		**By Wave 10**	
	No	Yes	No	Yes	No	Yes	No	Yes
**Vaping Status**								
Not-Reg Vaper	2,930 (82.5%)	583 (17.5%)	2,822 (80.1%)	691 (19.9%)	6,856 (94.7%)	278 (5.3%)	6,790 (93.2%)	344 (6.8%)
Reg Vaper	268 (79.7%)	86 (20.3%)	258 (75.4%)	96 (24.6%)	522 (87.7%)	68 (12.3%)	503 (85.3%)	87 (14.7%)
**Degree**								
No Degree	2,575 (84.4%)	474 (15.6%)	2,492 (81.5%)	558 (18.5%)	4,858 (93.3%)	233 (6.7%)	4,787 (91.4%)	304 (8.6%)
Has Degree	623 (74.1%)	195 (25.9%)	587 (72.6%)	230 (27.4%)	2,521 (95.6%)	112 (4.4%)	2,505 (94.7%)	128 (5.3%)

SUPPORTING INFORMATION APPENDIX E

Sensitivity analysis: vaping variable categorised as 0 = non-vaper, 1 = infrequent or regular vaper

Table S2. Estimates of Effects of SEP on Smoking Cessation/Relapse, With and Without Interaction by Vaping Status

	**Unadjusted Association between Having No Degree and Smoking Cessation/Relapse**	**Controlled Direct Effect of Having No Degree on Smoking Cessation/Relapse**	**Controlled Direct Effect of Having No Degree on Smoking Cessation/Relapse Among Non-Vapers**	**Controlled Direct Effect of Having No Degree on Smoking Cessation/Relapse Among Vapers**
	OR [95% CI]	OR [95% CI]	OR [95% CI]	OR [95% CI]
**Smoking Cessation**				
[Reference: Has Degree]				
No Degree	0.62 [0.52-0.73]	0.65 [0.54-0.77]	0.58 [0.48-0.72]	1.23 [0.70-2.14]
**Smoking Relapse**				
[Reference: Has Degree]				
No Degree	1.35 [1.06-1.75]	1.77 [1.38-2.25]	1.57 [1.10-2.25]	1.73 [0.93-3.21]

Notes: OR = odds ratio and 95% CI = 95% confidence interval. Vaping is defined as using or sometimes using e-cigarettes. The unadjusted association uses UKHLS wave 8 sampling weights to account for survey design and non-response but does not adjust for any confounders. The controlled direct effect uses inverse probability weighted marginal structural modelling to additionally adjust for exposure-outcome confounders and mediator-outcome confounders.

Table S3. Estimates of Effects of SEP on Vaping Among Current Smokers and Ex-Smokers

	**Unadjusted Association between Having No Degree and Vaping**	**Controlled Direct Effect of Having No Degree on Vaping**
	OR [95% CI]	OR [95% CI]
**Wave 8 Vaping (Ex-Smokers)**		
[Reference: Has Degree]		
No Degree	1.27 [1.03, 1.58]	1.67 [1.35, 2.06]
**Wave 8 Vaping (Current Smokers)**		
[Reference: Has Degree]		
No Degree	1.05 [0.82, 1.35]	1.03 [0.79, 1.37]

Notes: OR = odds ratio and 95% CI = 95% confidence interval. Vaping is defined as using or sometimes using e-cigarettes. The unadjusted association uses UKHLS wave 8 sampling weights to account for survey design and non-response but does not adjust for any confounders. The controlled direct effect uses inverse probability weighted marginal structural modelling to additionally adjust for exposure-outcome confounders and mediator-outcome confounders.

Table S4. Estimates of Effects of Vaping on Smoking Cessation/Relapse

	**Unadjusted Association between Wave 8 Vaping and Smoking Cessation/Relapse**	**Controlled Direct Effect of Wave 8 Vaping on Smoking Cessation/Relapse**
	OR [95% CI]	OR [95% CI]
**Smoking Cessation**		
[Reference: Not Vaper]		
Vaper	1.12 [0.94-1.35]	0.98 [0.76-1.27]
**Smoking Relapse**		
[Reference: Not Vaper]		
Vaper	3.10 [2.32-4.13]	3.27 [2.37-4.50]

Notes: OR = odds ratio and 95% CI = 95% confidence interval. Vaping is defined as using or sometimes using e-cigarettes. The unadjusted association uses UKHLS wave 8 sampling weights to account for survey design and non-response but does not adjust for any confounders. The controlled direct effect uses inverse probability weighted marginal structural modelling to additionally adjust for exposure-outcome confounders and mediator-outcome confounders.

SUPPORTING INFORMATION APPENDIX F

Sensitivity analysis: SEP based on NSSEC, categorised as 0 = management/professional, 1 = not management/professional

Table S5. Estimates of Effects of SEP on Smoking Cessation/Relapse, With and Without Interaction by Vaping Status

	**Unadjusted Association between NSSEC and Smoking Cessation/Relapse**	**Controlled Direct Effect of NSSEC on Smoking Cessation/Relapse**	**Controlled Direct Effect of NSSEC on Smoking Cessation/Relapse Among Non-Regular Vapers**	**Controlled Direct Effect of NSSEC on Smoking Cessation/Relapse Among Regular Vapers**
	OR [95% CI]	OR [95% CI]	OR [95% CI]	OR [95% CI]
**Smoking Cessation**				
[Reference: Management/Professional]				
Not Management/Professional	0.75 [0.63-0.89]	0.95 [0.76-1.18]	0.93 [0.71-1.23]	2.02 [0.80-5.13]
**Smoking Relapse**				
[Reference: Management/Professional]				
Not Management/Professional	0.96 [0.74-1.25]	1.25 [0.95-1.66]	1.15 [0.75-1.78]	2.06 [0.87-4.90]

Notes: OR = odds ratio and 95% CI = 95% confidence interval. Regular vaping is defined as vaping at least weekly. The unadjusted association uses UKHLS wave 8 sampling weights to account for survey design and non-response but does not adjust for any confounders. The controlled direct effect uses inverse probability weighted marginal structural modelling to additionally adjust for exposure-outcome confounders and mediator-outcome confounders.

Table S6. Estimates of Effects of SEP on Regular Vaping Among Current Smokers and Ex-Smokers

	**Unadjusted Association between NSSEC and Vaping**	**Controlled Direct Effect of NSSEC on Vaping**
	OR [95% CI]	OR [95% CI]
**Wave 8 Regular Vaping (Ex-Smokers)**		
[Reference: Management/Professional]		
Not Management/Professional	0.83 [0.66-1.04]	1.09 [0.85-1.39]
**Wave 8 Regular Vaping (Current Smokers)**		
[Reference: Management/Professional]		
Not Management/Professional	1.09 [0.77-1.54]	1.01 [0.65-1.57]

Notes: OR = odds ratio and 95% CI = 95% confidence interval. Regular vaping is defined as vaping at least weekly. The unadjusted association uses UKHLS wave 8 sampling weights to account for survey design and non-response but does not adjust for any confounders. The controlled direct effect uses inverse probability weighted marginal structural modelling to additionally adjust for exposure-outcome confounders and mediator-outcome confounders.

SUPPORTING INFORMATION APPENDIX G

Sensitivity analysis: SEP based on NSSEC, categorised as 0 = in paid employment, 1 = not in paid employment

Table S7. Estimates of Effects of SEP on Smoking Cessation/Relapse, With and Without Interaction by Vaping Status

	**Unadjusted Association between NSSEC and Smoking Cessation/Relapse**	**Controlled Direct Effect of NSSEC on Smoking Cessation/Relapse**	**Controlled Direct Effect of NSSEC on Smoking Cessation/Relapse Among Non-Regular Vapers**	**Controlled Direct Effect of NSSEC on Smoking Cessation/Relapse Among Regular Vapers**
	OR [95% CI]	OR [95% CI]	OR [95% CI]	OR [95% CI]
**Smoking Cessation**				
[Reference: in Paid Employment]				
Not in Paid Employment	0.80 [0.69-0.93]	0.86 [0.73-1.01]	0.88 [0.72-1.08]	1.62 [0.89-2.92]
**Smoking Relapse**				
[Reference: in Paid Employment]				
Not in Paid Employment	0.61 [0.49-0.77]	1.02 [0.81-1.30]	1.06 [0.74-1.53]	0.90 [0.41-2.03]

Notes: OR = odds ratio and 95% CI = 95% confidence interval. Regular vaping is defined as vaping at least weekly. The unadjusted association uses UKHLS wave 8 sampling weights to account for survey design and non-response but does not adjust for any confounders. The controlled direct effect uses inverse probability weighted marginal structural modelling to additionally adjust for exposure-outcome confounders and mediator-outcome confounders.

Table S8. Estimates of Effects of SEP on Regular Vaping Among Current Smokers and Ex-Smokers

	**Unadjusted Association between NSSEC and Vaping**	**Controlled Direct Effect of NSSEC on Vaping**
	OR [95% CI]	OR [95% CI]
**Wave 8 Regular Vaping (Ex-Smokers)**		
[Reference: in Paid Employment]		
Not in Paid Employment	0.46 [0.37-0.57]	0.67 [0.53-0.86]
**Wave 8 Regular Vaping (Current Smokers)**		
[Reference: in Paid Employment]		
Not in Paid Employment	0.87 [0.66-1.14]	0.70 [0.51-0.96]

Notes: OR = odds ratio and 95% CI = 95% confidence interval. Regular vaping is defined as vaping at least weekly. The unadjusted association uses UKHLS wave 8 sampling weights to account for survey design and non-response but does not adjust for any confounders. The controlled direct effect uses inverse probability weighted marginal structural modelling to additionally adjust for exposure-outcome confounders and mediator-outcome confounders.

SUPPORTING INFORMATION APPENDIX H

Sensitivity analysis: no qualifications used instead of no degree as the educational attainment SEP measure)

Table S9. Estimates of Effects of SEP on Regular Vaping Among Current Smokers and Ex-Smokers

	**Unadjusted Association between Having No Qualifications and Regular Vaping**	**Controlled Direct Effect of Having No Qualifications on Regular Vaping**
	OR [95% CI]	OR [95% CI]
**Wave 8 Regular Vaping (Ex-Smokers)**		
[Reference: Has Qualifications]		
No Qualifications	0.69 [0.54-0.87]	0.86 [0.67-1.11]
**Wave 8 Regular Vaping (Current Smokers)**		
[Reference: Has Qualifications]		
No Qualifications	1.17 [0.88-1.55]	1.03 [0.74-1.40]

Notes: OR = odds ratio and 95% CI = 95% confidence interval. Regular vaping is defined as vaping at least weekly. The unadjusted association uses UKHLS wave 8 sampling weights to account for survey design and non-response but does not adjust for any confounders. The controlled direct effect uses inverse probability weighted marginal structural modelling to additionally adjust for exposure-outcome confounders and mediator-outcome confounders.

Table S10. Estimates of Effects of SEP on Smoking Cessation/Relapse, With and Without Interaction by Regular Vaping Status

	**Unadjusted Association between Having No Qualifications and Smoking Cessation/Relapse**	**Controlled Direct Effect of Having No Qualifications on Smoking Cessation/Relapse**	**Controlled Direct Effect of Having No Qualifications on Smoking Cessation/Relapse Among Non-Regular Vapers**	**Controlled Direct Effect of Having No Qualifications on Smoking Cessation/Relapse Among Regular Vapers**
	OR [95% CI]	OR [95% CI]	OR [95% CI]	OR [95% CI]
**Smoking Cessation**				
[Reference: Has Qualifications]				
No Qualifications	0.67 [0.57-0.79]	0.74 [0.61-0.89]	0.75 [0.56-0.99]	0.30 [0.14-0.65]
**Smoking Relapse**				
[Reference: Has Qualifications]				
No Qualifications	0.63 [0.49-0.82]	0.99 [0.76-1.29]	1.14 [0.65-1.98]	2.13 [1.14-3.96]

Notes: OR = odds ratio and 95% CI = 95% confidence interval. Regular vaping is defined as vaping at least weekly. The unadjusted association uses UKHLS wave 8 sampling weights to account for survey design and non-response but does not adjust for any confounders. The controlled direct effect uses inverse probability weighted marginal structural modelling to additionally adjust for exposure-outcome confounders and mediator-outcome confounders.
